# Different gene-specific mechanisms determine the ‘revised-response’ memory transcription patterns of a subset of *A. thaliana* dehydration stress responding genes

**DOI:** 10.1093/nar/gku220

**Published:** 2014-04-17

**Authors:** Ning Liu, Yong Ding, Michael Fromm, Zoya Avramova

**Affiliations:** 1 University of Nebraska School of Biological Sciences, 1901 Vine Street, Lincoln, NE 68588, USA; 2 School of Life Sciences, University of Science & Technology of China, 443 Huangshang Road, Hefei, Anhui 230027, China; 3 University of Nebraska Center for Biotechnology and Center for Plant Science Innovation, 1901 Vine Street, Lincoln, NE 68588, USA

## Abstract

Plants that have experienced several exposures to dehydration stress show increased resistance to future exposures by producing faster and/or stronger reactions, while many dehydration stress responding genes in *Arabidopsis thaliana* super-induce their transcription as a ‘memory’ from the previous encounter. A previously unknown, rather unusual, memory response pattern is displayed by a subset of the dehydration stress response genes. Despite robustly responding to a first stress, these genes return to their initial, pre-stressed, transcript levels during the watered recovery; surprisingly, they do not respond further to subsequent stresses of similar magnitude and duration. This transcriptional behavior defines the ‘revised-response’ memory genes. Here, we investigate the molecular mechanisms regulating this transcription memory behavior. Potential roles of abscisic acid (ABA), of transcription factors (TFs) from the ABA signaling pathways (ABF2/3/4 and MYC2), and of histone modifications (H3K4me3 and H3K27me3) as factors in the revised-response transcription memory patterns are elucidated. We identify the TF MYC2 as the critical component for the memory behavior of a specific subset of MYC2-dependent genes.

## INTRODUCTION

Unable to escape unfavorable environmental conditions, plants respond to various stresses by coordinately activating physiological changes and by altering gene expression patterns to improve resistance and increase survival chances under stress (rev. in (1)). It has been reported that pre-exposure to a stress (high salinity, cold or high temperature) altered plants’ responses by increasing their resistance to a similar stress in future exposures (2–5). Pre-treatment (priming) with hormones (jasmonic acid, JA, abscisic acid, ABA, salicylic acid, SA or its synthetic analogue, benzothiadiazole *S*-methylester) increased the systemic immunity and induced stronger responses from responding genes upon subsequent treatments than displayed by non-primed plants (6–9). Plants that have experienced stress from water withdrawal also showed an improved capacity to tolerate subsequent water deficiency through adjustments leading to a decreased osmotic potential (10). In a repeated stress, a subset of the dehydrations stress responding genes provides transcriptional responses that are different from responses during the first stress (11,12). These observations have lead to the concept of ‘stress memory’, implying that during subsequent exposures plants provide responses that are different from their responses during the first encounter. Some memory effects may be transferred to the next generation, as observed for flagellin and ultraviolet radiation treatments (13). In general, stress memory may provide the benefits of enhanced resistance and/or protection against biotic and abiotic stresses (14), although some pre-treatments may result in increased sensitivity to deleterious effects, as demonstrated by grapevines exposed to ozone in consecutive years (15).

By producing transcripts at a different level upon a repeated encounter with the same stress, the memory genes alter the levels of encoded proteins, presumably, enabling plants to meet the challenges of recurring stresses. The molecular mechanisms associated with stress memory are largely unknown but the altered gene expression indicates that a ‘memory response’ to a subsequent similar stress is more complex than repetitive activation of the same response mechanism.

Our operational criterion for memory is that transcript levels from dehydration stress responding genes in a second stress (S2) must be different from the levels produced during the first stress (S1) (12,16). In a genome-wide transcriptome analysis of repeatedly stressed Arabidopsis plants, we have identified 1963 genes that provided altered responses to a subsequent stress (16). These genes define the dehydration stress ‘transcriptional memory genes’; 4616 genes providing similar responses upon each stress represent the ‘non-memory’ dehydration stress response category (16).

Depending on the level of transcripts produced in a S2 compared with the levels in the first stress (S1), four distinct transcriptional memory response-patterns were recognized (16). These were designated as [+/+], [+/−], [−/−], [−/+] memory categories, where the first sign indicates transcript levels in S1 compared with W: (+) if higher, (−) if lower; the second sign indicates transcript levels in S2 relative to S1. Similar responses during each exposure were displayed by 2177 up-regulated and 2439 down-regulated genes representing the [+/=] and [−/=] non-memory gene categories, respectively (16). Among the memory genes, 362 genes induced in S1 produced significantly higher transcript levels in S2 (W < S1 < S2) constituting the [+/+] memory category; 857 genes, denoted as the [+/−] memory genes, displayed higher transcript levels in S1 than the pre-stressed levels in W, but in S2 transcript levels were significantly lower than in S1 (W < S1 > S2). This type of transcriptional responses revealed existence of a novel class of transcription memory genes that robustly increase transcription in S1, but after returning to the base-level in recovery do not respond, or provide only a weak response, to a second stress. Because their transcript levels in S2 remain closer to the initial pre-stressed watered (W) levels that were significantly lower than in S1, we refer to them as ‘revised-response’ [+/−] memory genes (16).

Gene ontology (GO) analysis of whole genome-transcriptome data has indicated a biased functional distribution among the memory types suggesting possible biological relevance of transcriptional memory patterns (16). About 30% of the [+/−] memory genes encode proteins regulating osmotic pressure, water balance and wall modifications implicated in plants’ stress responses and environmental adaptation, while 32% of the [+/−] memory genes are co-regulated with various other stress/hormone signaling pathways (for extended analyses see (16)). By robustly increasing transcript levels in S1, but returning to pre-stressed (W) levels in S2, the [+/−] memory genes re-adjust protein levels of metabolic, water and ion transport proteins under repeated dehydration stress playing, thus, a critical role in maintaining cellular homeostasis. The large number of [+/−] memory genes implicated in responses to other abiotic stress- and hormone-signaling pathways (16) suggest that, although many genes shared by multiple response systems get activated in S1, they are no longer engaged in subsequent responses to dehydration stress. Consequently, the transcriptional interactions between the various response networks will be different during a second dehydration stress from the interactions occurring in the first. Presumably, transcriptional memory provides the benefits of reducing the costs of transcribing genes that are required for stress responses other than dehydration (17).

Here, we investigate molecular mechanisms that determine, or contribute to, the transcriptional behavior of [+/−] ‘revised-response’ memory genes using a few genes as a model. The genes were randomly chosen from the whole-genome data to represent a broad interval of transcriptional responses in S1 and were used initially to verify transcriptome [+/−] patterns (16). They belong in the most highly represented functional categories encoded by the revised response [+/−] memory genes implicated in overlapping biotic and abiotic signaling pathways and in plasma membrane-wall associated functions in Arabidopsis. These include genes for the transcription factors (TFs) RAP2.4, RAP2.6 and ABR1 (18–20), the *AT1G51780* gene encoding ILL5 from the auxin signaling pathway (21), *AT3G28220* encoding a TARF-like protein, a putative regulator of anthocyanin biosynthesis, the *AT3G25760*, *AT2G06050* and *AT2G46370* genes involved in the synthesis of jasmonic acid and the biologically active jasmonyl–isoleucine conjugate (AOC1, OPR3 and JAR1), respectively, (22,23), as well as for the JA-responsive genes *AT4G08870* (Arginase) and *At1g19180* (JAZ1). The [+/−] memory genes *At2g36830* and *At1g26770* encode, respectively, the aquaporin GAMMA-TIP1.1 (24) and the cell wall-loosening protein expansin10 involved in stress relaxation/extension of plant cell walls (25) and formation of nematode-induced syncytia in roots (26).

Here, we examine the potential roles of chromatin (histone H3 Lys4, H3K4me3, and Lys27, H3K27me3) modifications in the [+/−] memory responses of *Arabidopsis thaliana* genes, as well as of the plant hormone ABA and of TFs involved in the ABA-dependent regulatory pathways (26–28). The phytohormone ABA and the TFs from the ABF and the MYC2/MYB2 families are key mediators in dehydration stress signaling and much of the ABA signaling pathway has been elucidated (30–34).

The three leucine zipper ABA-binding factors, ABFs (AREB1/ABF2, AREB2/ABF4 and ABF3) are major components of the ABA-signaling pathways regulating a large number of dehydration stress response genes (34). The bHLH transcription factor MYC2 is a positive regulator of ABA signaling and is considered a master regulator of the crosstalk between ABA and the other hormone signaling pathways (2,35). Importantly, MYC2 negatively regulates its own expression by directly binding the *MYC2* promoter and, after reaching a critical threshold levels, triggering a negative autoregulatory loop (36,37).

Despite the considerable amount of data available on the roles of ABA, of AREB1/ABF2, AREB2/ABF4, ABF3 and MYC2 in plants’ responses to dehydration stress, their potential roles as memory factors in responses to recurring stresses is less known. Here, we elucidate their potential involvement in the memory behavior of [+/−] revised response genes.

## MATERIALS AND METHODS

### Plant growth and treatments

Wild-type *A. thaliana* plants (Col-0) and various mutant backgrounds analyzed here were grown in potting soil in growth rooms at 22°C with a 12-h light photoperiod and light intensity of 180 μmol/m^2^/s. Dehydration stress and full watered recovery were performed on 3-week-old seedlings as previously described with some modifications (12,16). After removing the plants from soil and washing any remaining soil from their roots, plants were placed in humid chambers overnight to recover from potential root wounding during extraction from soil and to exclude possible effects on the transcriptional responses. Transcript levels measured in rosette leaves collected from recovered plants before initiating stress treatments are designated as pre-stressed (W) levels. The S1 treatment is achieved by exposing plants to dry air for 90 min, followed by recovery (R1), achieved by placing plants in humid chambers for 22 h with their roots in a few drops of water. For a subsequent stress treatment, R1 plants were gently blotted onto filter paper to remove water and air-dried for 90 min (S2) followed by a recovery (R2). The same procedures were repeated for S3, R3 and S4. Knockout *myc2* and *35S::MYC2* overexpressing lines were kind gifts from Dr John Browser from (Washington State University), the *areb1/areb2/abf3* triple mutant line was kindly provided by Dr Kazuko Yamaguchi-Shinozaki (The University of Tokyo). The *aba2/gin1–3* mutant line was obtained from ABRC (CS6147).

### Reverse transcription and real-time PCR

Total RNA isolation and reverse transcription with oligo(dT)_15_ primer (C1101, Promega) were performed as described previously (38). The amounts of individual genes were measured with gene-specific primers by real-time polymerase chain reaction (PCR) analysis with a Cycler IQ Real-time PCR Instrument (Bio-Rad) and SYBR Green mixture (Bio-Rad). The relative expression or amount of specific genes was quantitated with the 2^−ΔΔ*C*t^ calculation (39), according to the manufacturer's software (Bio-Rad), where ΔΔ*C*t is the difference in the threshold cycles and the reference housekeeping gene, which was ubiquitin for expression analyses or relative to input DNA for chromatin immunoprecipitation (ChIP) assays. All primers were evaluated to ensure robust amplification. The standard curves and amplification efficiency are shown in Supplementary Figure S1. The specific primers used are shown in Supplementary Table S1.

**Figure 1.**
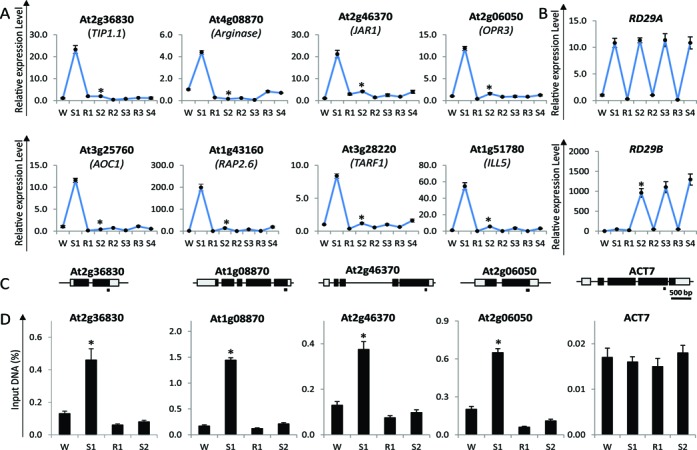
Transcription patterns of Arabidopsis genes in response to multiple stresses and Ser2 Pol II levels during two consecutive dehydration stress treatments. (**A**) Transcript levels of genes from the [+/−] memory category randomly chosen from the genome-wide transcriptome data measured by real-time quantitative RT-PCR. *UBQ10* was used as an internal control. (**B**) Transcript levels for the *RD29B* [+/+] memory, and for the *RD29A* [+/=] non-memory, genes. (**C**) Schematic diagram of the genes, with the promoter region (to left), the 5′- and 3′-untranslated regions (grey box), exons (dark box), and introns (thin lines between exons). The regions analyzed by chromatin immunoprecipitation with specific antibodies and real-time qPCR (ChIP–qPCR) are indicated with a bar under the genes. (**D**) Ser2P Pol II levels measured during the W, S1, R1 and S2 phases of the stress/recovery cycle as indicated. The results with *ACT7* are shown as an internal control. Experiments were repeated at least three times, each with three replicates, and the representative experiment shown indicates the mean ± SEM, *n* = 3 replicates. Asterisks indicate statistical significance of transcript levels in S1 and S2 based on Student's *t*-test. *P < 0.01.

The raw transcriptome sequence files for W, S1 and S3 have been uploaded, together with gene expression result files, to NCBI's Gene Expression Omnibus under sequence number GSE48235.

### Chromatin immunoprecipitation assay

The ChIP assay was performed accord­ing to the previously described method (12,40,41). The specific antibodies (1:150 dilution) used for Ser2P Pol II (ab5095, Abcam, Cambridge, MA, USA, Lot: 703307); trimethyl-H3K4 (ab1012, Abcam, Lot: GR561731-1); trimethyl-H3K27 (#07-449, Millipore, Lot: JBC1924326) or H3 (ab1791, Abcam, Lot: 517990) were used. Purified DNA was analyzed by real-time PCR with gene-specific primers shown in Supplementary Table S2.

## RESULTS

### Revised response memory genes

In whole-genome transcriptome analyses of transcripts from W non-stressed plants and from plants subjected to one or three dehydration stresses by consecutive exposures to dry air (for 2 h), each followed by watered recovery of 22 h periods, 857 genes displayed significantly increased transcript levels in S1 but in subsequent stresses of similar duration and strength, the transcript levels were significantly lower than in S1 (16). These genes define the [+/−] revised-response memory category (16). The transcription patterns measured by qRT-PCR of the genes used in this study confirm the patterns obtained from the whole genome transcriptome (Figure 1A; Supplementary Figure S2) and illustrate the signature transcription profiles of [+/−] ‘revised-response’ memory genes: elevated transcript levels in S1, lowered transcript levels during watered recoveries (R1/R2/R3) but low, or no, responses during subsequent exposures (S2/S3/S4). The transcription patterns of the [+/+] memory response gene, *RD29B* and of the non-memory [+/=] gene, *RD29A* (Figure 1B) are also included to illustrate the remarkable differences in the transcriptional behavior of three distinct response-types to repeated stress. Despite the fact that all genes were induced in S1, their transcription in S2 was either super-induced (in the [+/+] memory category), not induced (in the [+/−] memory category), or induced to the same degree as in S1 (the [+/=] non-memory category).

The most dramatic differences in the memory responses of all memory genes are displayed between the first and the second stress treatments. As the transcriptional responses of the [+/−] memory genes to three or more successive stresses (in S3 or S4) are similar to those in S2 (Figure 1A) and to avoid excessive stress treatments, from here on we analyze the transcriptional responses at four phases of the treatment cycle: the initial phase (W), after the first stress (S1), after watered recovery (R1), and after a second exposure (S2).

### Distribution of the elongating polymerase II

Whether the transcript levels displayed by the [+/−] memory genes during the four phases of the treatment were regulated by transcription was examined by measuring the polymerase II phosphorylated at serine 2 of CTD (Ser2P Pol II) levels at the 3′-ends of the genes. Active transcription correlates with accumulation of higher amounts of the elongating Ser2P Pol II at transcribed genes (12,42). ChIP assays with anti-Ser2P Pol II antibodies and with specific primers overlapping regions towards the 3′-ends of analyzed [+/−] memory genes (Figure 1C), established Ser2P Pol II levels were higher in S1 and low in W, R1 and S2 (Figure 1D). In comparison, Ser2P Pol II levels at the constitutively expressed housekeeping gene *ACT7* remained constant.

Earlier, we found that Ser2P Pol II levels and the amounts of transcripts produced from the memory *RD29B* and the non-memory *RD29A* genes reflect the transcription rates from these genes (12). Therefore, the transcription patterns of the examined [+/−] memory genes are regulated at the level of transcription and the low transcript levels in W, R1 and S2 correlate with low transcription. Despite experiencing stress conditions similar to S1, transcription in S2 is not induced, illustrating the signature transcriptional response feature of the [+/−] revised-memory genes.

### Chromatin marks at the [+/−] memory genes

**Figure 2.**
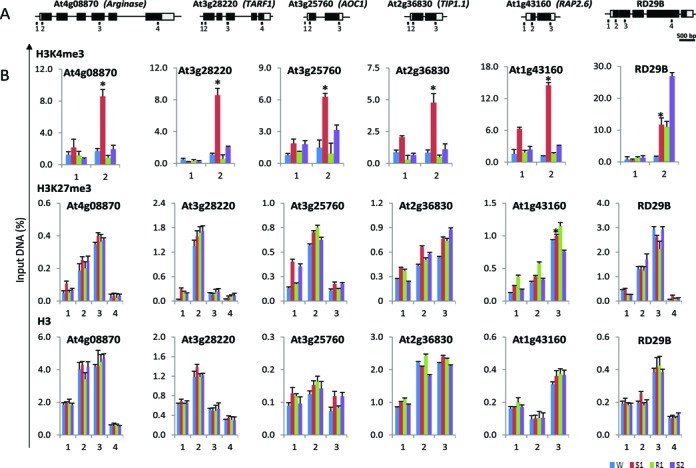
Levels of H3K4me3, H3K27me3 and histone H3 along the [+/−] ‘revised-response’ memory genes during four for different phases of the treatment cycle. (**A**) Schematic diagram of analyzed genes. Annotations are as described in Figure 1C, above. The regions analyzed by ChIP-qPCR are indicated by the numbered bars below. (B) The amounts of specific modifications and of histone H3 measured at the regions annotated along analyzed genes in non-stressed watered (W, blue), singly stressed (S1, red), recovered (R1, green) and secondly stressed (S2, purple) plants. Experiments were repeated at least three times, each with three replicates, and the representative experiment shown indicates the mean ± SEM, *n* = 3 replicates. Asterisks indicate statistical significance of S1 and S2 based on Student's *t*-test. *P < 0.01.

H3K4me3 marks are associated with active transcription (43) serving as a ‘memory mark’ for the [+/+] *RD29B* and *RAB18* genes (12). The operational definition for a memory mark is that it must last longer than the stimulus and should affect subsequent transcriptional behavior (12). To establish whether H3K4me3 plays a role in the transcriptional behavior of the [+/−] genes, ChIP assays were performed with H3K4me3-specific antibodies and primers at the 5′-ends of tested genes (43,44) (Figure 2A). High H3K4me3 levels accumulated in S1, but were low in W, R1 and S2 (Figure 2B), correlating with the transcript levels measured at all phases of the treatment cycle. Nucleosome levels are constant (Figure 2B, bottom row) supporting the conclusion that highly H3K4me3 modified nucleosomes at the [+/−] memory genes are present only in S1. Therefore, the H3K4me3 levels at the [+/−] memory genes correlate with their transcriptional activity in a stark contrast with *RD29B*, where high H3K4me3 retained during lower transcription in (R1) remain as a ‘memory’ from the previous transcription in S1 (Figures 1B and 2B).

Whether H3K27me3 serves as a repressive memory mark for the low transcription in R1 and S2 was examined next. As no specific peak accumulation of H3K27me3 has been defined along gene sequences in Arabidopsis (45), ChIP assays with H3K27me3-specific antibodies were performed with primers that overlap several positions along the genes (Figure 2A). Remarkably, no significant changes in H3K27me3 levels were measured for all tested genes, regardless of whether transcription was high (in S1) or low (in W, R1 and S2) (Figure 2B). Even at the superinduced [+/+] memory gene *RD29B* and the non-memory *RD29A* gene the H3K27me3 levels during active transcription remained unchanged from their levels in the transcriptionally less active states (Figure 2B; Supplementary Figure S3). Collectively, the results indicate that presence of H3K27me3 at the dehydration stress response genes did not suppress induction of transcription.

Lack of significant differences in H3K27me3 levels at the memory and non-memory genes, or during transcriptionally active/inactive gene states indicated H3K27me3 was not an epigenetic ‘memory’ mark for the examined dehydration stress responding genes.

### Abscisic acid and the [+/−] memory

The role of ABA in dehydration stress responses is well established but its potential role as a memory factor is less known. It is important to emphasize that endogenous ABA levels repetitiously increase upon each dehydration stress but return to low levels during watered recovery and that the levels in S2 are similar to the levels in S1 (12). Thereby, a model, wherein retention of a high ABA level from the previous stress was responsible for the super-induced transcription of the [+/+] memory genes, is unlikely (12). Whether endogenous ABA plays a role in the transcriptional behavior of [+/−] memory genes was examined here by using *aba2* mutant plants deficient in ABA biosynthesis (46).

**Figure 3.**
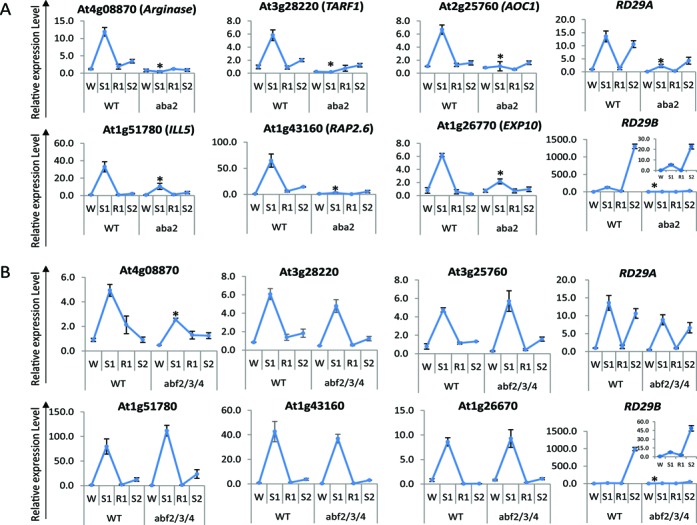
Role of ABA and of the ABA-dependent transcription factors, AREB1/ABF2, AREB2/ABF4 and ABF3, in the memory behavior of [+/−] ‘revised-response’ memory genes. (**A**) Transcript levels of [+/−] memory genes in the wild type and in *aba2* mutant backgrounds during the four stress/recovery periods, as indicated. (**B**) Transcript levels of [+/−] memory genes in the wild type and in *areb1/areb2/abf3* triple mutant backgrounds. Transcript levels from the non-memory *RD29A*, and the [+/+] memory *RD29B* genes are shown for comparison. Transcript levels for *RD29B* in *aba2* and *areb1/areb2/abf3* mutant backgrounds are shown in a different scale (insets). *UBQ10* was used as an internal control. Results are the average of three independent experiments, each with two replicates. Error bars indicate the standard error of the mean. Asterisks indicate statistical significance of different transcript levels in S1 between Col-0 and the mutant backgrounds based on Student's *t*-test. *P < 0.01.

Among 14 tested [+/−] memory genes, 11 significantly reduced S1 transcript levels in the *aba2* background (Figure 3A, Supplementary Figure S4), one (*At5g64750*) was de-repressed, and two were not significantly affected (Supplementary Figure S4). Thereby, [+/−] genes have a different dependence on ABA for their induction in S1. We conclude that the mechanisms responsible for increased transcription in S1 are gene-specific and that both ABA-dependent and ABA-independent pathways are involved in the [+/−] memory behavior.

### The ABA-regulated ABRE-binding TFs do not determine the [+/−] memory behavior

The ABFs AREB1/ABF2, AREB2/ABF4 and ABF3, well-known as mediators of the ABA-signaling pathway (34) were examined here for a potential involvement in the memory behavior of ABA-dependent [+/−] revised response genes. Effects on their expression were measured in a triple loss-of-function (*areb1/areb2/abf3*) mutant background. No statistically significant changes in transcript levels in S1 were measured from the ABA-dependent [+/−] memory genes, except for the slight decrease in *At4g08870* transcripts (Figure 3B). Expression of *RD29A* and *RD29B*, shown as controls (Figure 3B), are in agreement with known effects of the ABFs on their transcription (12,34). The results suggest AREB1, AREB2 and ABF3 are not the main factors inducing transcription of the ABA-dependent [+/−] genes in S1 or for their memory-response patterns during the subsequent stress (S2).

### MYC2 is critical for the memory of specific [+/−] revised-response genes

The finding of the *MYC2* gene among the [+/−] ‘revised-response’ memory genes in the transcriptome datasets (16) and its known role in regulating dehydration stress response genes (27,47,48) suggested a possible involvement in the memory responses of the [+/−] memory genes analyzed here.

**Figure 4.**
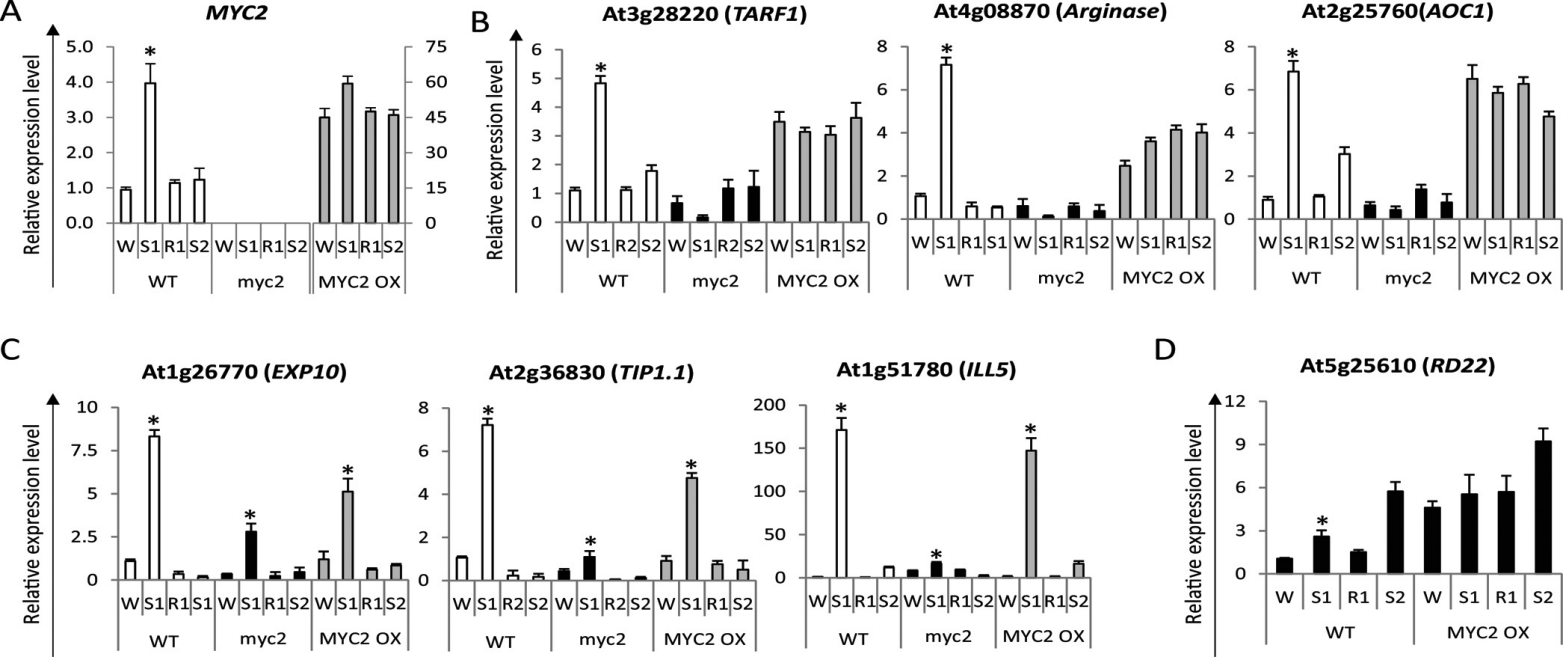
Role of MYC2 in the memory behavior of [+/−] ‘revised-response’ memory genes. (**A**) Transcript levels of *MYC2* in wild type, *myc2* and *35S:: MYC2* backgrounds. (**B** and **C**) Transcript levels of the six ABA-dependent, AREB1/AREB2/ABF3-independent [+/−] memory genes (see Figure 3, above) analyzed in wild type, *myc2* mutant and *MYC2*-overexpressing backgrounds illustrating all six [+/−] genes require MYC2 for optimal expression in S1; (B) [+/−] ‘revised-response’ memory genes that have lost memory responses in MYC2-overexpressing background, displaying high transcript levels in all four treatment states in the absence of stress; (C) [+/−] ‘revised-response’ memory genes that depend on MYC2 for expression in S1 but are not affected by the constitutive presence of MYC2 in the overexpressing background. (**D**) The signature MYB2-dependent gene, *RD22*, is induced in S2, despite low *MYB2* levels in wild type. Overexpression of *MYB2* raises *RD22* transcription irrespective of stress signals. *UBQ10* was used as an internal control. Experiments were repeated three times, each with three replicates, and the representative experiment shown indicates the mean ± SEM, *n* = 3 replicates. Asterisks indicate statistical significance between S1 and S3 in wild type, myc2 mutant and *MYC2* overexpressing lines, respectively. Statistical analysis was performed based on Student's *t*-test. *P < 0.01.

The [+/−] memory transcription pattern of *MYC2* in response to repeated dehydration stress exposures (confirmed by qRT-PCR, Figure 4A) was consistent with the negative autoregulation of the *MYC2* gene (36,37,49) as well as with the regulation of MYC2 protein activity by proteolysis (50). The question, most relevant for our studies, however, was whether MYC2 was the ‘memory factor’ determining the memory responses of MYC2-dependent [+/−] dehydration stress genes.

To address this question we identified, first, [+/−] memory genes that depend on MYC2 for their induced transcription in S1. Among the 14 [+/−] memory genes examined in the loss-of-function *myc2* background 10 genes decreased significantly transcription in the *myc2* mutants (Figure 4B and C, SF5), two were strongly de-repressed and two were not significantly influenced (SF5). We conclude that MYC2 is not a general TF responsible for the increased transcription in S1 of all [+/−] memory genes.

The second question was whether the [+/−] memory genes that depend on MYC2 in S1 depend on MYC2 also for their memory response in S2. However, the negative autoregulation of the *MYC2* gene (the low *MYC2* expression in S2, in particular) left unclear its potential involvement in the low-transcription memory responses of regulated genes in S2. To eliminate the negative regulatory loop and to examine the role of MYC2 in the memory responses of targeted genes in S2, we analyzed their behavior in the presence of constitutively expressed MYC2 (in the 35S*::MYC2* overexpressing background (51)) (Figure 4A). Among the genes found to require MYC2 for activation in S1 (Figure 4B and C), three genes (*TARF1, Arginase* and *AOC1*) lost their memory in the MYC2-overexpressing background and produced transcripts in S2 at levels similar to those S1 (Figure 4B). Moreover, the expression of these genes in W and in R1 was also increased indicating that, in addition to their memory behavior, MYC2 acted as a general activating TF for these genes capable of inducing their transcription in the absence of stress signals.

Importantly, the memory responses of the other three genes in S2 were not affected by the constitutively expressed MYC2 (Figure 4C). Thereby, although MYC2 was required for the induction of their transcription in S1, MYC2 was not regulating their memory responses in S2 (see below).

### MYC2 does not determine the [+/−] memory behavior of all MYC2-dependent genes

The inability of MYC2 to induce the expression of dependent genes in S2 in the *MYC2* overexpressing background indicated that low transcription from these genes did not result from the low *MYC2* levels in S2. We conclude that different mechanisms regulate the memory responses of the [+/−] memory genes even when the same TF regulates their responses in S1. Moreover, the [+/−] memory behavior of *MYC2* does not pre-determine the transcriptional behavior of regulated genes under repeated stress even for genes that are directly regulated by MYC2. The most striking example is the *RD22* gene, a marker MYC2-regulated gene that binds MYC2 to its promoter and elevates its transcription under dehydration stress (27,52). In agreement, we found *RD22* was up-regulated in S1 and overexpression of MYC2 caused constitutively high transcription even in the absence of a stress (Figure 4D). Remarkably, however, *RD22* was up-regulated also in S2, despite low *MYC2* levels in the wild type suggesting that a different factor(s) activate *RD22* in S2.

We conclude that different molecular mechanisms are involved in the transcriptional responses of the response genes in a single stress and when responding to repeated stress exposures.

## DISCUSSION

The four distinct transcriptional response patterns displayed by Arabidopsis genes upon multiple exposures to dehydration stress uncovered a new level of complexity of the transcription regulatory mechanisms. Sustained hormone levels, accumulation of specific TFs, and epigenetic mechanisms have been proposed as factors determining the memory behavior (12,47,53–60). Chromatin-based mechanisms, involving histone acetylation/deacetylation as well as histone H3 and DNA methylation, have been considered as epigenetic regulators of stress-responding genes (56–60). As emphasized above, we distinguish between a chromatin mark (a modification that is dynamically associated with a process but is removed at the conclusion of that process) and an epigenetic (memory) mark implying persistence longer than the initial stimulus establishing the mark. Importantly, a histone modification must affect the subsequent transcriptional performances of the gene in order to meet the criterion for a memory mark. In this context, H3K4me3, whose levels correlate with dynamically changing transcription of the [+/−] memory genes (Figures 1 and 2B) do not meet the criterion for a memory mark. In contrast, the higher H3K4me3 levels retained at the [+/+] memory gene *RD29B* during R1 when transcription is low (Figure 2B) illustrates the idea of a ‘memory mark’ from the previously active (S1) state (12). Accumulation of H3K4me3 on the nucleosomes of defense-response genes upon chemical priming before their induction by a pathogen attack also provides an epigenetic mark in the salicylic acid signaling pathway (8,54).

Repressed transcription in S2 of the [+/−] memory genes suggested the plausibility that H3K27me3 might play a role in this process. H3K27me3 is considered a silencing mark counter-balancing the activating functions of H3K4me3 in both animal and plant developmentally regulated genes (61–63). The function of H3K27me3 at genes that dynamically change transcription in response to environmental stresses is substantially less known (64,65). The H3K27me3 levels at each position along the dehydration stress memory genes (Figure 2B), or the non-memory *RD29A* gene (Supplementary Figure S3C), during actively transcribed states revealed no significant changes from baseline ‘high’ H3K27me3 levels present during the initial pre-stressed (W) conditions, when expression from all tested genes is at their basal (low) level. These observations indicated that H3K27me3-modified nucleosomes of dehydration stress response genes did not prevent efficient transcription by RNA polymerase II or H3K4me3 accumulation upon induction of [+/−]. Moreover, high H3K27me3 levels were present even when transcription was superinduced, as observed with [+/+] memory genes (Figure 2B, (40)).

Collectively, the data indicate that H3K4me3 and H3K27me3 co-exist and function independently during the transcription of dehydration stress-responding memory genes. This is in contrast to the general assumption that the presence of H3K4me3 and H3K27me3 is mutually exclusive and that the two marks play opposite roles in genes’ activity (66,67). However, these results support the intriguing possibility that H3K27me3 plays different roles when regulating the expression of developmental genes and of genes that alter expression rapidly in response to environmental conditions (40). As H3K27me3 levels did not change, regardless of whether the genes were transcribed or not, we conclude H3K27me3 does not function as an epigenetic memory mark for the examined dehydration stress responding genes.

Although synthesis of ABA is critical for the induction of transcription in S1 of specific [+/−] memory genes (Figure 3A, Supplementary Figure S4), transcription of these genes was not triggered in S2, despite the presence of endogenous ABA in S2 at similar levels as in S1 (12). Most likely, then, a different mechanism regulates the response in S2. We propose that in S1, ABA is needed to activate/deactivate-specific factor(s) that will execute the memory response in S2. In addition, as not all [+/−] memory genes depend on the ABA signal for their transcription in S1 (Supplementary Figure S4), the results indicate existence of an ABA-independent memory mechanism for the revised-response transcriptional behavior.

Two main types of ABA-dependent regulatory pathways have been described in plants (68). One, mediated by the basic leucine zipper/ABA-responsive element (bZIP/ABRE) system (29) and a second, mediated by ABA via MYC/MYB transcription factors inducing drought-responsive genes in Arabidopsis (28,49). Our results revealed that the ABA-dependent TFs ABRE1/ABRE2/ABF3 were not essential for the induction of the [+/−] memory genes in S1 or for their memory behavior in S2 (Figure 3B). Instead, MYC2 was found to mediate the ABA-dependent responses in S1 for some, but not all, tested [+/−] memory genes (Figure 4B and C; Supplementary Figure S5). Moreover, not all [+/−] memory genes that require MYC2 for transcription in S1 depended on MYC2 for their responses in S2. Therefore, MYC2 is the ‘memory factor’ only for specific [+/−] memory genes. These genes lose their memory responses in S2 (Figure4B) and display high transcript levels even in the absence of stress (in W and R1) when *MYC2* is constitutively expressed (Figure 4A),

The versatile roles of MYC2 as an activator and as a repressor of genes involved in the ABA, JA and auxin signaling pathways (35–37,47,50–52) have been considered in the context of *MYC2*'s early expression in the presence of signaling molecules (JA or ABA), its negative autoregulation (27,36), and the regulation of its activity by phosphorylation and proteolysis (50). The temporal correlation between MYC2 protein accumulation and its opposite effects on wound-responsive and pathogen-responsive genes was used to explain its differential transcriptional activity with these genes (50).

The negative autoregulatory loop regulating *MYC2* transcription is consistent with a model wherein transcription in S1 is required to achieve repression in S2. MYC2 protein is present longer than 24h post-induction (50) supporting a lasting inhibitory effect upon the transcription of the *MYC2* gene and offering a mechanistic explanation for its own [+/−] transcriptional patterns in S1 and S2. The transcriptional patterns of the [+/−] revised-response genes *At4g08870*, *At3g28220* and At3g25760 (Figure 4B), then, occur as a consequence of the auto-suppressed transcription of MYC2. It is important to emphasize that this model does not require the genes to be regulated directly by MYC2. Thus, although all examined here [+/−] genes that depend on MYC2 to elevate transcription in S1 carry the G-box (CAC(G/A)TG) motifs (providing potential binding sites for the transcription factor), only a specific subset among them depends on MYC2 for the memory response in S2. These genes lose memory in S2 and are constitutively expressed in *35S::MYC2* overexpressing plants. These effects could be achieved either directly, by MYC2 binding to the promoter, or via another transcription factor that translates the effects of MYC2.

A strong supporting argument is provided by the signature MYC2-regulated gene, *RD22*. Although *RD22* increases its transcription in response to dehydration stress through the binding MYC2 to the G-box in its promoter (27,28,52)*, RD22* is strongly induced again in S2 when the levels of *MYC2* are low (Figure 4D). Therefore, a direct binding of MYC2 to the *RD22* promoter is not sufficient to ensure a [+/−] memory response in S2.

Among the most important conclusions of this study is that the transcriptional behavior of a TF cannot be used to explain, or predict, the transcriptional behavior of its target genes under repeated stresses, even when the genes depend on the TF for their responses in the first stress, or when the TF binds directly to the promoter of the genes. We have identified 73 genes (7%) of the *A. thaliana* [+/−] memory genes that encode TFs, most of them broadly shared among multiple abiotic and hormone response networks (16). While the memory behavior of some TFs will be determining the memory responses of specific genes, as found for a specific subset of the MYC2-dependent genes, it is not possible to predict the memory behavior of all their targets as the TF memory behavior is not the general mechanism imparting the memory transcriptional patterns to all regulated genes.

Collectively, our data have indicated that even genes belonging in the same memory category are regulated by diverse and gene-specific mechanisms (as shown here and in (40)) compounding the task of elucidating these mechanisms. Uncovering these mechanisms will be critical for understanding how plants cope and adapt under a changing environment.

## Accession Number

NCBI's Gene Expression Omnibus under sequence number: GSE48235.

## SUPPLEMENTARY DATA

Supplementary Data are available at NAR Online.

SUPPLEMENTARY DATA
