# Supplementary material for: Different gene-specific mechanisms determine the ‘revised-response’ memory transcription patterns of a subset of A. thaliana dehydration stress responding genes
Source: Nucleic Acids Res. 2014 Apr 17;42(9):5556–66. doi: 10.1093/nar/gku220 (PMC4027201; doi:10.1093/nar/gku220)
Supplement: SUPPLEMENTARY DATA [file supp_42_9_5556__index.html]

Different gene-specific mechanisms determine the ‘revised-response’ memory transcription patterns of a subset of A. thaliana dehydration stress responding genes — Different gene-specific mechanisms determine the ‘revised-response’ memory transcription patterns of a subset of A. thaliana dehydration stress responding genes — SUPPLEMENTARY DATA 

# Different gene-specific mechanisms determine the ‘revised-response’ memory transcription patterns of a subset of *A. thaliana* dehydration stress responding genes

## SUPPLEMENTARY DATA

**Files in this Data Supplement:**

- SUPPLEMENTARY DATA
