# Supplementary material for: Different gene-specific mechanisms determine the ‘revised-response’ memory transcription patterns of a subset of A. thaliana dehydration stress responding genes
Source: Nucleic Acids Res. 2014 Apr 17;42(9):5556–66. doi: 10.1093/nar/gku220 (PMC4027201; doi:10.1093/nar/gku220)
Supplement: SUPPLEMENTARY DATA [file supp_gku220_nar-00002-h-2014-File006.pdf]

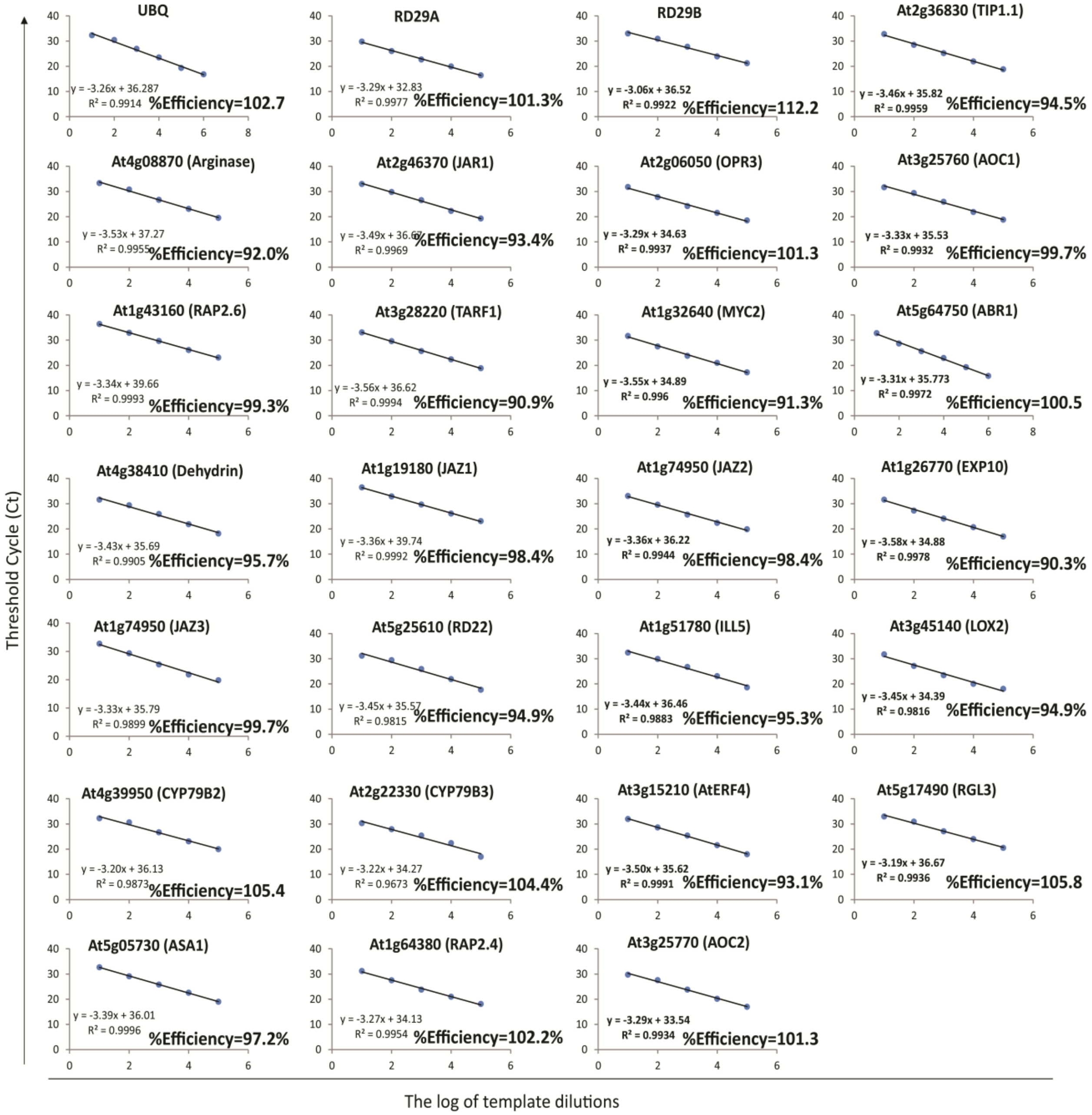

Supplementary Figure 1 Evaluation of the efficiencies of the primers used in quantitative real-time PCR experiments. Standard curve with the threshold cycle (Ct) plotted against the log of the starting quantity of each dilution. Each dilution was assayed in triplicate. The equation for the regression line, the r value, and the calculated amplification efficiency (%Efficiency) are shown on the graph.

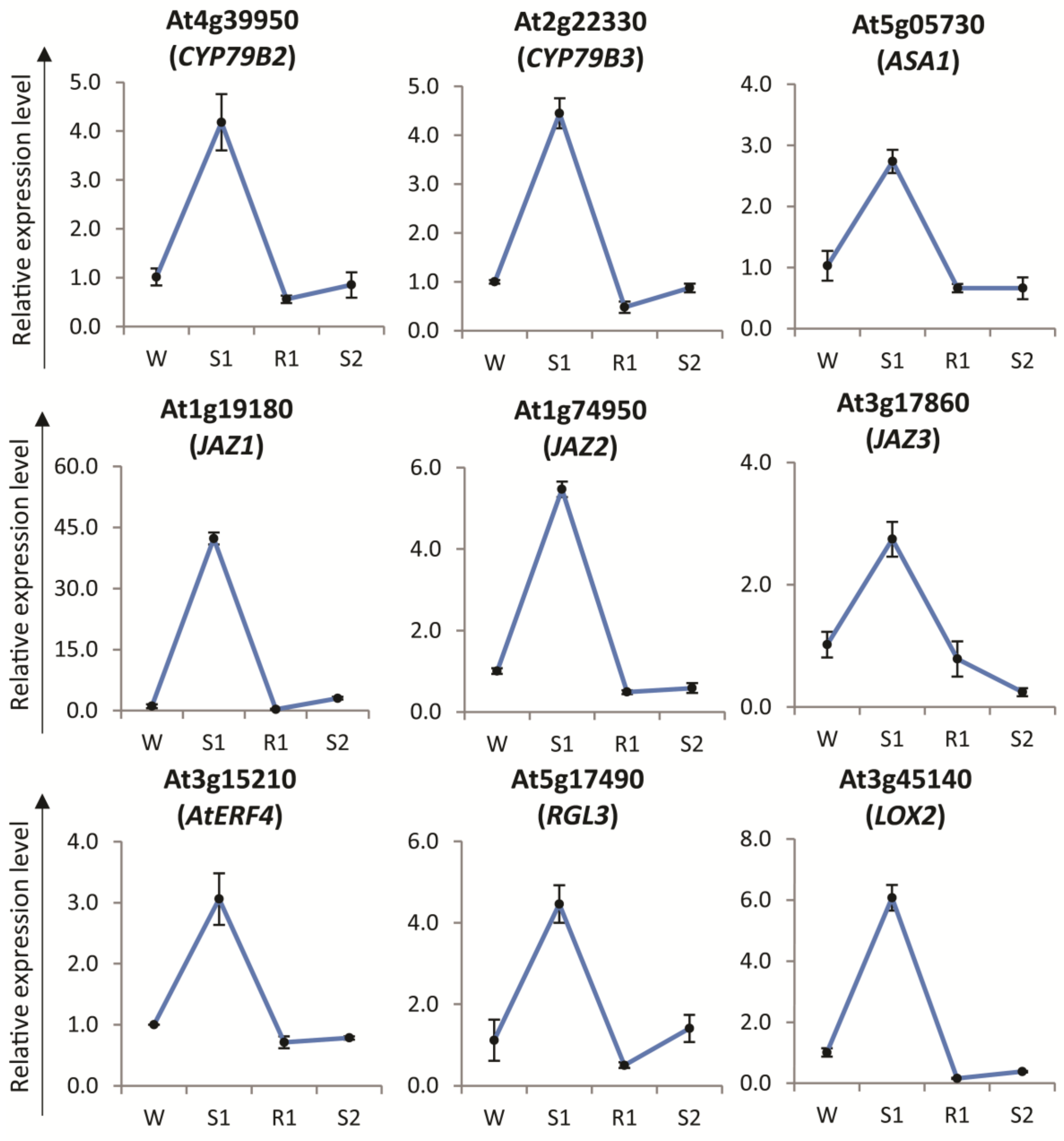

Supplementary Figure 2. Transcription patterns of additional Arabidopsis genes in response to two consecutive dehydration stress treatments.
Transcript levels of additional [+/-] memory category genes measured by real-time quantitative RT-PCR. *UBQ10* was used as an internal control. Results are the average of three independent experiments, each with two replicates. Error bars indicate the standard error of the mean.

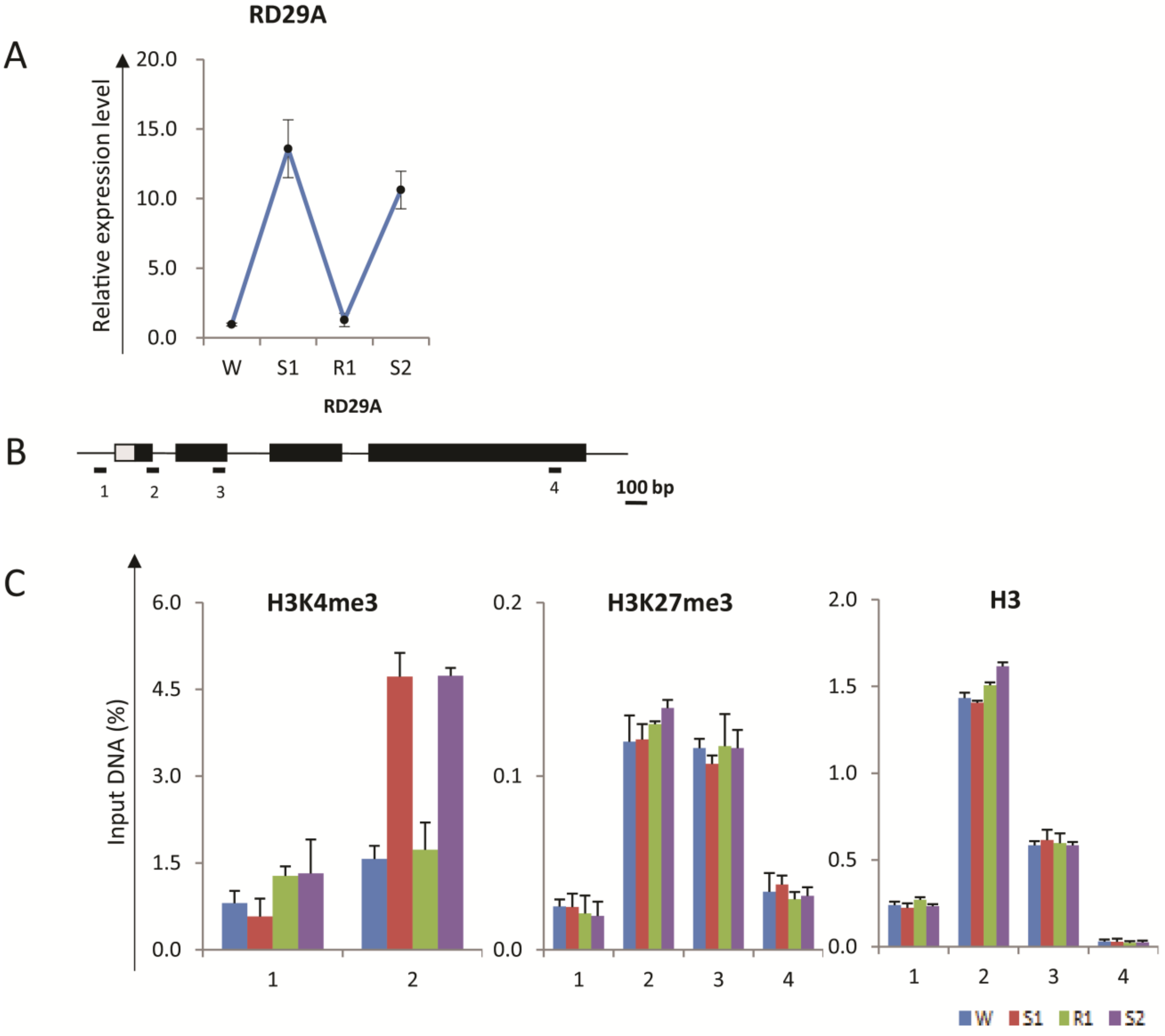

Supplementary Figure 3. Transcription, H3K4me3, and H3K27me3 levels of *RD29A* in response to repeated dehydration stress.

A) Transcript levels of *RD29A*; **B)** schematic presentation of *RD29A*. Numbered bars under the gene indicate regions recovered with specific primers in the ChIP assay; **C)** amounts of recovered DNA in the ChIP assays with specific antibodies, as indicated at locations along the gene sequence indicated by the numbered bar in (B). Experiments were repeated at least three times, each with three replicates, and the representative experiment shown indicates the mean ± s.e.m., *n* = 3 replicates.

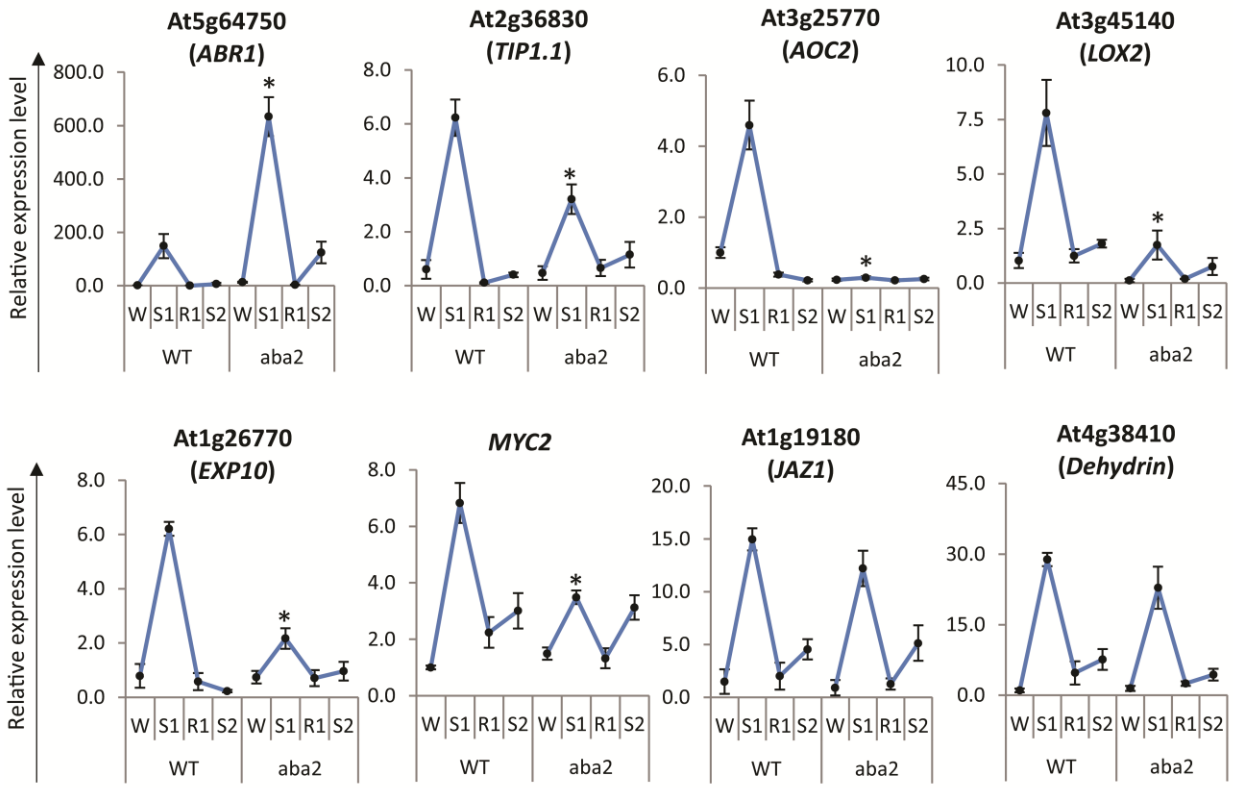

Supplementary Figure 4. Transcript levels of additional [+/-] memory genes in the *aba2* background
Results are the average of three independent experiments, each with two replicates. Error bars indicate the standard error of the mean. Significant changes in transcript levels in S1 in wild type and *aba2* backgrounds are marked by asterisks. Statistical significance is based on Student's t-test. *, $p<0.01$.

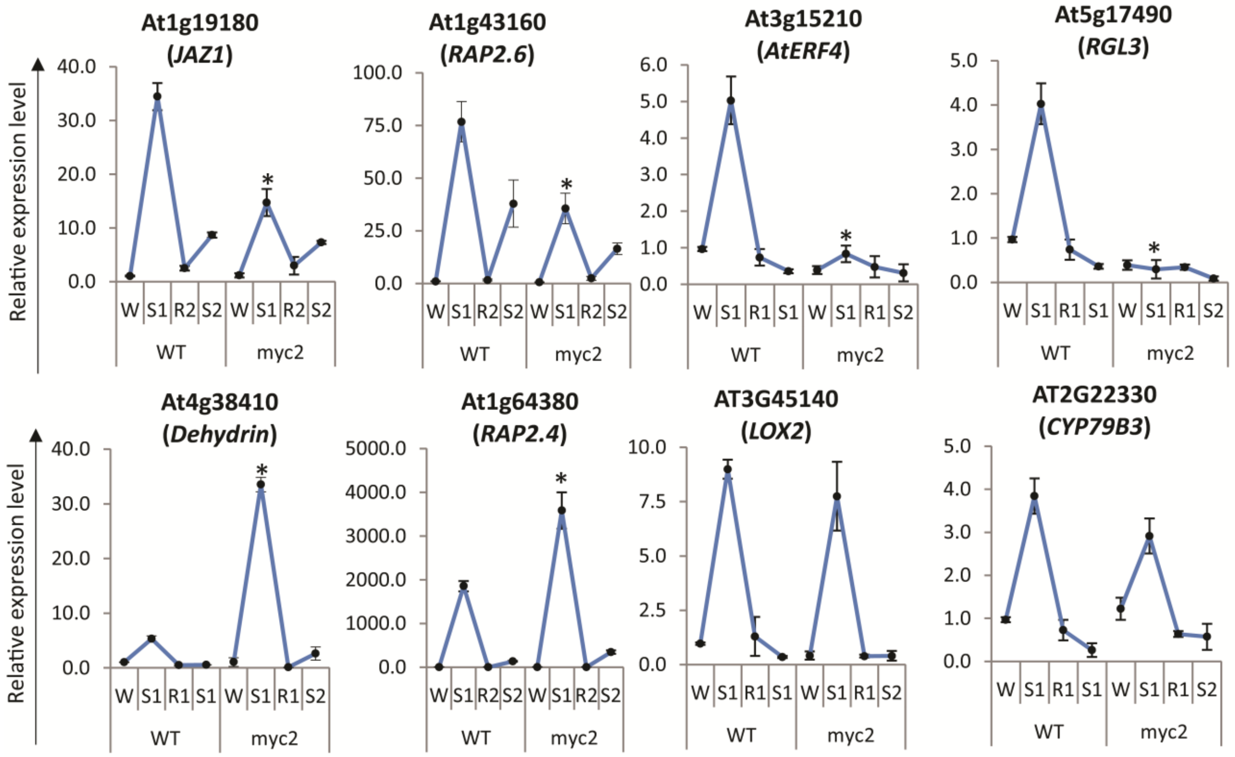

Supplementary Figure 5. Transcript levels of [+/-] memory genes in the *myc2* background
Results are the average of three independent experiments, each with two replicates. Error bars indicate the standard error of the mean. Significant changes in transcript levels in S1 in wild type and *aba2* backgrounds are marked by asterisks. Statistical significance is based on Student's t-test. *, $p<0.01$.

Supplementary Table 1: List of primers used for qRT analysis

Genes	Sequence (5'→ 3')
AT5G52310 (RD29A)	CAGATGGTGAGAAAGCGAAA
	CCCTACTTTGTGGCATCCTT
AT5G52300 (RD29B)	ACGAGCAAGACCCAGAAGTT
	AGGAACAATCTCCTCCGATG
AT2G36830 (TIP1.1)	CTGGACATGGACCAACCACT
	CTCGTGTGTGGTGTTGATGA
AT4G08870(Arginase)	AACAAGGCAAGAGGTTTGGA
	TACACGCCCTTCACTCCTTC
AT2G46370 (JAR1)	ATTGCAACTGTTTCGCACTG
	GGACACCTCCCGTTGATATG
AT2G06050(OPR3)	GGAAGCAGTTCACGCTAAGG
	CCGAGATTGGTTTGTTCGTT
AT3G25760(AOC1)	AATACCGAAAACCCCAGACC
	CGAGATCTCCGAGACCAAAC
AT1G43160(RAP2.6)	TCTTTGCCTCCTCAACCATT
	TGGTCGTTGCCTTACTCCTC
AT3G28220(TARF1)	TGTTGCGGTTGATGTTAAGC
	AATCCCCAACTGTTTGCTTG
AT1G51780(ILL5)	ACATTCCGAGCCCTTTTACC
	GCTTGCTTGCGTTGTGATAA
AT1G32640(MYC2)	GCGTTGATGGATTTGGAGTT
	TTGCTCTGAGCTGTTCTTGC
AT5G64750(ABR1)	AGGAGAGAGGTGGAGGAAGG
	CCCGGACCTGACATGTTACT
AT4G38410(Dehydrin)	GGTCACAATGGAAAGCCTGA
	TCATTGGTATGACCAGGAAGC
AT1G19180 (JAZ1)	GCATGCAAGCCTGATGTCAAT

	TGCCTAGGAAACAGATTCGTC
At1g26770 (EXP10)	TTTTTCAACGCATTGCTCAG
	TTCCTCCTCTTCTCCTGCAA
At3g25770 (AOC2)	CTCTCTCCCAAAACGGGAAT
	TCCGAGACCGAACATTAAGC
AT1G64380(RAP2.4)	AAGAGGCAATGATGGGAGTG
	TGGCAAGAACTTCCCAAATC
AT5G25610(RD22)	GCGATTCGTCTTCCTCTGAT
	CTCCGCCTTTACCTACTTGG
AT4G05320 (UBQ10)	AGGATGGCAGAACTCTTGCT
	TCCCAGTCAACGTCTTAACG
At4g39950 (CYP79B2)	ACAAGGCAACCCATTGCTTA
	CTCCGGTTTGTTCACCATCT
At2g22330 (CYP79B3)	ACCGCTGATGAAATCAAACC
	CGTTTGATGGGTTGTCTGG
At5g05730(ASA1)	CATCGAACGTTATTCCCATGT
	GTCCCAGCAAGTCAAACCAT
At1g74950 (JAZ2)	TCGGTTCCTTGAGAAGAGGA
	CTGAGCCAAGCTGGGTTAGT
At3g17860 (JAZ3)	AGCATCCCTGGCTAGGTTTT
	CTGCGACAATCTGTCGATGA
At3g15210 (AtERF4)	TCTCGAGCTGAGTGACCAGA
	GTCGGAGGAGAAGCACAGTC
At5g17490 (RGL3)	AAGCGAACCATAACGGTGAC
	AACACCATCTTCGAGCGAGT
At3g45140 (LOX2)	AACTACGATTGCATGGGTCA
	TCGGTTGGGAAAGTATCCTC

Supplementary Table 2: List of primers used for ChIP-qPCR experiments

Genes	Regions	Sequence (5'-->3')
AT4G08870	Region 1	CACCGACGAAAAGAAAGGTC
		AGATCTTGTGCTTATGGATGGA
	Region 2	ATGTGGAAGATTGGGCAGAG
		GCATCAATGACACGGTTCTG
	Region 3	GGAGGACCCGTGGATATTCT
		GCATAGCCACCTTCCATGAT
	Region 4	CAACCTTCAGGGAGATTTGG
		TTTTGACATTTTTGCGGCTA
AT3G28220	Region 1	ATCTGGTCGCAATCCATCTT
		GGATCTTTTGTGCGTTCGAT
	Region 2	GCTTGGCTTGCTTCATTTCT
		TATGGGAGGACATTGGGCTA
	Region 3	CTAATCCCAATGCTGGTTGG
		ATTTGTAAAGGCGGTCATGC
	Region 4	CAAGCAAACAGTTGGGGATT
		TTCAAATTCAATCTCCATCTTCAA
AT3G25760	Region 1	ACCGGCCAAAAGTACATCAA
		ATGGGTCACCAACGAATGAT
	Region 2	TTGAGCAATGGCTTCTTCTACA
		CCAAGAAGAGAGCTTCGATGA
	Region 3	GATTTGCCACTGGAGCTCAT
		ACAGGACACGAGAAAGATAAGACT
AT2G36830	Region 1	TATCCGCCACGTAGATTGGT
		TTTGATTGGAGCGGTTAGAGA
	Region 2	TCATGCCGATCAGAAACATC
		GGCGACGACAAAGATCAAAG
	Region 3	CTGGACATGGACCAACCACT

		CTCGTGTGTGGTGTTGATGA
AT1G43160	Region 1	TGGCTAAATCAATCAACGTGTC
		CATGTCTTTCTCTAGCCGTCA
	Region 2	TACCACCGCAATTTCAAACA
		CCGGTAATCAATGGTTGAGG
	Region 3	TGCAAAACTCTATGCAACCTTC
		TGGCTGCAACATGCCTACTA
AT5G52310 (RD29A)	Region 1	ACCGACATCAGTTTGAAAGAAA
		TGGTGTGACGTCAAAGTCATT
	Region 2	AGGAACCACCACTCAACACA
		CACCATTCTCATGATGTTCAACT
	Region 3	TCACTAAACATGGACAAAGCAA
		TGCATCGATCACTTCAGGTT
	Region 4	CTCCATCAAGAAGCCATGAA
		GGCGAATACTCGTTTCTTCC
AT5G52300 (RD29B)	Region 1	CGTAGAGAGCAACTGGCTGA
		ATTCTGACACGTACGATGCG
	Region 2	GAGACAGAAGCCAGAGAGAGG
		TTCAAGTGAATCAATCAAACCC
	Region 3	AATGGAGTCACAGTTGACACG
		GGATGGTGAATTCTGATTGGT
	Region 4	TGGAAGTGACGGTTGAGAAG
		ACCGCTCCTTTAACTTTCCC
AT5G09810 (ACT7)	Region 3	GCTGACCGTATGAGCAAAGA
		GATCCTCCGATCCAGACACT
